# Analysis of the current situation and influencing factors of night shift nurses’ sense of occupational benefit

**DOI:** 10.1097/MD.0000000000040539

**Published:** 2024-11-15

**Authors:** Zhenfan Liu, Xiaoting Yan, Cui Chen, Jijun Wu, Jing Lu

**Affiliations:** a Department of Nursing, Deyang People’s Hospital, Deyang, China; b Department of Nursing, Sichuan Nursing Vocational College, Chengdu, China.

**Keywords:** night nurses, nurses, psychological resilience, sense of occupational benefit, shift nurses

## Abstract

The purpose of this study was to investigate the current status and influencing factors of night shift nurses’ sense of occupational benefit, and to explore the influence of psychological resilience on the sense of occupational benefit. 2022 from August to October, a cross-sectional survey was conducted on 719 night shift nurses using a general information questionnaire, psychological resilience scale, and sense of occupational benefit scale. A total of 719 valid questionnaires were collected. The score of night shift nurses’ sense of occupational benefit was (137.40 ± 21.10) and psychological resilience score was (65.63 ± 17.75). Age (β = 3.359, *P* < .05) was significantly correlated with sense of occupational benefit. Education (β = 3.586, *P* < .05) was significantly correlated with the sense of occupational benefit, and whether or not they had participated in outbreak prevention and control (β = −2.321, *P* < .05) was significantly correlated with the sense of occupational benefit. Similarly, psychological resilience (β = 0.859, *P* < .05) was significantly associated with the sense of occupational benefit. Night shift nurses’ sense of occupational benefit was moderate to high, and interventions should be taken to enhance the sense of occupational benefit based on nurses’ age, education, whether they have participated in epidemic prevention and control, and psychological resilience.

## 1. Introduction

Currently, with the improvement of social and economic conditions, patients’ medical needs are changing accordingly. Patients are no longer simply to treat the disease for the purpose, but also need medical personnel to provide them with more humanistic care and medical services, which puts forward higher requirements for medical personnel.^[1]^ Nurses, as the main body of medical personnel, not only need to complete high-intensity clinical nursing work, but also need to continuously strengthen their own learning, effectively improve professional competence and work quality to adapt to the changes in the industry and society.^[2]^

However, the changing needs of patients have not improved the allocation of human resources for nurses. In China, as of 2022, although there are more than 5.2 million registered nurses in the country, the number of registered nurses per 1000 population is only about 3.7, which is seriously lower than 15.7 per 1000 population in the United States, 13.5 in Germany, and 12.7 in Japan. The long history of low manpower allocation and high workload has made nurses need to bear more work pressure.^[3]^ Studies have shown that for every 1 nurse less staffed, adverse events reported by nurses will increase by 1.04 times.^[4]^ At the same time, due to the influence of traditional concepts, most nurses are often just regarded as a part attached to doctors, and are identified as only being able to engage in some simple clinical care work, without their own independent professional value and role, making their professional recognition and social status low; in addition, due to the influence of social status, the group of nurses under the high workloads are not rewarded by the corresponding reasonable pay, and compared with the doctors and the other industry employees, the economic income of nurses is also at a low level, making it difficult for nurses to perceive the benefits and value of their own work, which results in a decline in the motivation of nurses to work, making them prone to the manifestation of burnout.^[5]^

Previous studies have shown that^[6]^ the overall prevalence of burnout among nurses globally is as high as 11.23%, with about 1 in 10 nurses showing high levels of burnout; a meta-analysis of the detection rate of burnout among medical personnel in China also showed that the overall burnout detection rate among Chinese medical personnel was as high as 65.57%.^[7]^ Burnout has also been proven to be a serious problem in China. And burnout has also been shown to be closely related to nurses’ willingness to leave their jobs.^[8]^ Related reports show that^[9]^ 22.02% of nurses in China often have the idea of changing jobs, which is a great challenge to the supply of service quality and the stability of human resources. Therefore, it is crucial to increase the professional value of nurses, enhance their motivation, reduce their negative emotions, and mitigate their willingness to leave their jobs.

As a positive psychological variable, occupational sense of benefit mainly refers to the fact that nurses can feel the gains and benefits of their work from the clinical nursing work they are engaged in, and recognize that their work can promote their all-round development.^[10]^ Research has found that^[11]^ the sense of occupational benefit is an important mechanism to stimulate nurses’ inner potential, and a positive sense of occupational benefit can effectively enhance nurses’ sense of well-being at work, thus reducing burnout; at the same time, when nurses perceive the value of their work, the more they are willing to actively engage in it, creating efficient work performance, which directly reduces nurses’ willingness to leave and ensures the stability of human resources.^[12]^ At this stage, most surveys on nurses have focused on the nurse population as a whole, with less attention paid to the long night shift nurses, who play an important but often overlooked role in the healthcare team. Their dedication and contribution is vital to the proper functioning of healthcare organizations.^[13]^ It is well known that due to the scarcity of human resources during night shift hours, night shift nurses face a different work environment and work pressure than daytime nurses, as they are required to maintain a high level of alertness during the night, deal with emergencies, as well as potentially face higher work intensity and less support from their coworkers. This particular work environment may have a unique impact on nurses’ occupational psychology. At the same time, due to the reversal of daily work schedule and sleep quality, long-term night shift work may have an impact on nurses’ physiological and psychological state, such as sleep disorders, fatigue, and psychological stress, which makes most of the nurses reluctant to engage in night shift nursing, making it difficult for them to truly discover the value of their work and self-development in the night shift work, and thus making them prone to negative burnout, which will seriously affect the work efficiency and quality output.^[14–16]^ However, less research has been conducted on what level night nurses’ sense of occupational benefit is at the bottom and the factors that may reduce their sense of occupational benefit.

Psychological resilience, as an important evaluation index of mental health, mainly refers to the quality of an individual’s ability to adjust his or her emotions and behaviors from negative events or experiences and to integrate smoothly into the environment, which has a long-term protective effect on the individual’s development.^[17]^ According to the dynamic model of psychological resilience, it can be found that when an individual acquires external resources it will promote the accumulation of internal resources, which effectively promotes their own development.^[18]^ Studies have shown that psychological resilience, as an individual protective resource, can motivate nurses to grow out of their work dilemmas and focus on positive emotions, thus experiencing a sense of pride and accomplishment in their work; at the same time, nurses with higher levels of psychological resilience tend to have a stronger personal knowing ability to maintain positive attitudes toward their work, thus having higher levels of job satisfaction, and thus lowering the level of burnout.^[19,20]^ Theoretically, psychological resilience can enhance the level of career sense of benefit by acquiring job satisfaction.

Currently, there is no research on the factors influencing night shift nurses’ sense of occupational benefit and the relationship between psychological resilience and sense of occupational benefit in China. For these reasons, we focused our study on the special group of night shift nurses to investigate the factors related to occupational sense of benefit and to explore the relationship between psychological resilience and occupational sense of benefit of night shift nurses, in order to provide a reference for managers to develop targeted interventions to improve the occupational sense of benefit of night shift nurses, and to provide high-quality nursing services to patients.

## 2. Materials and methods

### 2.1. Study design and participants

This study is a cross-sectional research design, August–October 2022, 719 night shift nurses were conveniently selected as the participants of this survey. The sample size calculation formula, set = 0.05, based on the pre-survey results of the total standard deviation of night shift nurses’ sense of occupational benefit score = 21.01, it is hoped that the allowable error is not more than 2. The control value for this study was set at 0.5, which yielded = 424, and the sample size was approximately 509, taking into account 20% of the invalid questionnaires. Inclusion criteria were nurses who had obtained a license to practice nursing, had worked in clinical nursing for ≥1 year, and had ≥1 night shift per week. Participants were asked to fill out the questionnaire via WeChat on their cell phones. It took approximately 15 minutes to complete the questionnaire. To ensure the quality of the questionnaire, participants who took <3 minutes or more than 30 minutes to answer were excluded. The study was reviewed and approved by the Ethics Committee of Deyang People’s Hospital (No. 2020-04-141), and all respondents gave informed consent and voluntarily participated in this study. Our study adheres to the Helsinki Declaration by the WMA.

### 2.2. Methodology

#### 2.2.1. Survey instruments

##### 2.2.1.1. General information questionnaire

The questionnaire was determined by the research team after reviewing and discussing the literature, including gender, age, education, marital status, department, hospital level, average monthly income of an individual, title, years of working experience, and whether or not whether or not they have worked in the fight against the coronavirus disease 2019 (COVID-19).

##### 2.2.1.2. Sense of occupational benefit scale

The Sense of Occupational Benefit Scale was developed by Chinese scholars Hu et al,^[21]^ which was mainly used to measure the level of nurses’ sense of occupational benefit, with a Cronbach α coefficient of 0.958.^[22]^ Applied it to clinical nurses, and measured the Cronbach α coefficient of 0.968. The scale consists of positive occupational perception (7 items), good nurse-patient relationship (6 items), team belongingness (6 items), family and friend identification (6 items), and a positive sense of family and friend identification (6 items). The scale consisted of 33 items in 5 dimensions: positive career perception (7 items), good patient-nurse relationship (6 items), team belonging (6 items), family and friends’ identity (6 items), and self-growth (8 items). A 5-point Likert scale was adopted, with scores ranging from 1 to 5 from “strongly disagree” to “strongly agree.” The total score ranged from 33 to 165, and the higher the total score, the stronger the sense of occupational benefit. The reliability of the scale was good, with a Cronbach α coefficient of 0.893.

##### 2.2.1.3. Psychological resilience scale

Compiled by Connor KM and Davidson (2003) in 2002,^[23]^ which is mainly used to measure the psychological resilience of individuals. In 2005, Yu and Zhang^[24]^ revised the scale, and the Cronbach α coefficient was 0.910. Meng et al^[25]^ applied it to nurses, and the Cronbach α coefficient was 0.836. The scale consists of 25 items in three dimensions: resilience (13 items), strength (8 items) and optimism (4 items), and is composed of 25 items in three dimensions. The scale consisted of 25 items in 3 dimensions, and was rated on a 4-point Likert scale from “never” to “always,” with scores ranging from 0 to 4, respectively. The total score was 0 to 100, and the higher the score, the better the psychological resilience. The reliability of this scale was good, with a Cronbach alpha coefficient of 0.925.

### 2.3. Statistical analysis

The results were exported directly from the questionnaire star and analyzed using SPSS 25.0 statistical software, using descriptive analysis, *t* test, one-way ANOVA, Pearson’s correlation analysis, and multivariate hierarchical regression analysis to explore the variable relationships. Differences were considered statistically significant at *P* < .05.

## 3. Results

### 3.1. Night shift nurses’ sense of occupational benefit and psychological resilience score

A total of 719 night shift nurses were surveyed, and the results showed that the score of night shift nurses’ sense of occupational benefit was 137.40 ± 21.10, and the score of psychological resilience was 65.63 ± 17.75, and the scores of specific entries, see Table 1.

**Table 1:** Night shift nurses’ sense of occupational benefit and psychological resilience score (n = 719, *x̄±s*).

Sports event	Entry	Scoring range	Score
Sense of occupational benefit	33	33–165	137.40 ± 21.10
Positive career perception	7	7–35	28.65 ± 4.98
Nurse–patient relationship	6	6–30	25.69 ± 4.01
Recognition by family and friends	6	6–30	24.56 ± 4.25
Team affiliation	6	6–30	24.85 ± 4.08
Self-growth	8	8–40	33.65 ± 5.27
Psychological resilience	25	0–100	65.63 ± 17.75
Tough and durable	13	0–52	33.60 ± 9.65
Pessimistic	4	0–16	14.13 ± 3.06
Force	8	0–32	21.90 ± 5.64

### 3.2. Comparison of occupational benefit scores of night nurses with different characteristics

The results of this study showed that the occupational benefit scores of night nurses with different genders, hospital levels, ages, education, years of working experience, titles, and whether or not they have worked in the fight against the COVID-19 were different (*P* < .05), and for the detailed results, see Table 2.

**Table 2:** Comparison of occupational benefit scores of night nurses with different characteristics (*x* ± s).

Sports event	Number of persons	Sense of occupational benefit	*t*/*F* value	*P*-value
Distinguishing between the sexes			‐2.680	.009
Male	62	131.84 ± 16.63		
Women	657	137.93 ± 21.41		
Hospital level			4.981	.007
three-tier	363	138.68 ± 20.86		
Three-tier	230	138.31 ± 18.38		
Category B	126	132.06 ± 25.35		
Age (years)			35.479	.000
20 to <30	308	130.09 ± 20.47		
30 to <40	376)	142.81 ± 20.25		
>40	35	143.66 ± 15.83		
Academic qualifications			‐6.623	.000
Polytechnic	233	130.09 ± 22.82		
Undergraduate and above	486	140.91 ± 19.29		
Marital status			1.479	.135
Married	411	138.42 ± 20.18		
Unmarried	308	136.04 ± 22.23		
Monthly personal income ($)			1.618	.199
0 to <5000	325	136.07 ± 22.06		
5000 to <10,001	324	138.03 ± 19.81		
≥10,001	70	140.64 ± 22.16		
Working experience			12.908	.000
0 to <5	266	131.41 ± 20.83		
5 to <10	244	139.44 ± 20.07		
10 to <20	198	142.51 ± 21.06		
>20	11	145.27 ± 17.32		
Title			3.825	.022
Junior ranking	446	135.71 ± 21.25		
Middle level (in a hierarchy)	240	140.20 ± 21.14		
High level	33	139.97 ± 16.34		
Sections			2.223	.109
General medicine	355	138.99 ± 21.13		
Neurosurgery	241	135.32 ± 22.06		
Acute and critical illness	123	136.91 ± 18.75		
Whether or not they have worked in the fight against the COVID-19			6.037	.000
Be	303	142.78 ± 19.77		
Clogged	416	133.49 ± 21.20		

### 3.3. Correlation matrix analysis of psychological resilience and sense of occupational benefit among night shift nurses

The results of this study showed that the total psychological resilience score and the scores of each dimension were positively correlated with the total score of the sense of occupational benefit and the scores of each dimension (*R* = 0.537–0.984, all *P* < .01) The correlation between psychological resilience and the sense of occupational benefit among night shift nurses is shown in Table 3.

**Table 3:** Correlation matrix analysis of psychological resilience and sense of occupational benefit among night shift nurses (*n*, *r*).

Sports event	1	2	3	4	5	6	7	8	9	10
1. Sense of occupational benefit	1									
2. Positive career perception	0.943*	1								
3. Nurse-patient relationship	0.887*	0.836*	1							
4. Family and friends approval	0.916*	0.809*	0.709*	1						
5. Team Attribution	0.951*	0.881*	0.795*	0.860*	1					
6. Self-growth	0.961*	0.859*	0.814*	0.888*	0.902*	1				
7. Psychological resilience	0.767*	0.752*	0.632*	0.722*	0.745*	0.721*	1			
8. Tough.	0.738*	0.720*	0.596*	0.708*	0.712*	0.696*	0.984*	1		
9. Power	0.786*	0.770*	0.677*	0.714*	0.760*	0.740*	0.969*	0.922*	1	
10. Optimism	0.679*	0.675*	0.537*	0.643*	0.680*	0.625*	0.915*	0.858*	0.872*	1

* *P* < .01 correlation is significant at the .05 level (2-tailed).

### 3.4. Multivariate stratified regression analysis: the effect of psychological resilience on perceived occupational benefit

Stratified regression analyses were used to explore the factors influencing night shift nurses’ sense of occupational benefit, with sense of occupational benefit as the dependent variable, and variables meaningful for univariate analyses of variance (gender, hospital level, age, education, years of service, job title, and Whether or not they have worked in the fight against the COVID-19), and psychological resilience as the independent variables for regression analyses. The independent variables were included in 2 steps. The main general characteristics significantly different from the sense of occupational benefit were included as control variables in the first stratum. Psychological resilience was included as a test predictor variable in the second level. For detailed assignments, see Table 4. In order to test for the presence of multicollinearity between the variables, multicollinearity analysis was used at each step, and the results showed that the tolerance values between the independent variables were all >0.1, and the variance inflation factors were all <3, which showed that there was no serious multicollinearity between the independent variables.

**Table 4:** Assignment of independent variables.

Sports event	Assignment method
(A person’s) age	1 = 20 to <30 years old; 2 = 30 to <40 years old; 3 = >40 years old
Level of hospital	1 = Level IIIA hospital; 2 = level IIIB hospital; 3 = level II hospital
Highest level of education	1 = Specialist; 2 = Bachelor’s degree and above
Title	1 = Beginner; 2 = Intermediate; 3 = Advanced
Whether or not they have worked in the fight against the COVID-19	1 = Yes; 2 = No
Years of experience	1 = 0 to <5 years; 2 = 5 to <10 years; 3 = 10 to <20 years; 4 = >20 years
Distinguishing between the sexes	1 = Male; 2 = female
Psychological resilience	Substituting the original value

In Model 2, the psychological resilience variable was added, and the results of the study showed that psychological resilience was a significant predictor of night shift nurses’ sense of occupational benefit (β* = *0.859, *P *< .001), independently explaining 45.0% of the sense of occupational benefit.

## 4. Discussion

Overall, night shift nurses’ sense of occupational benefit score was (137.40 ± 21.10), which was moderate to high and lower than Liu et al’s^[12]^ study of nurses supporting Wuhan during the COVID-19 pandemic, but higher than the results of Fang et al’s^[26]^ study of all nurses. Previous studies have shown that nurses with higher frequency of night shifts have lower sense of occupational benefit, but the current study of night shift nurses was different from previous ones, and the sense of occupational benefit was higher than that of the survey of nurses as a whole.^[27]^ The reasons were analyzed: on the one hand, due to the lack of human resources, most of the night shift nurses in China care for the patients in the whole ward alone, although the heavy workload and the reversed work and rest schedules of day and night will increase the nurses’ work pressure, they must have solid theoretical knowledge and professional skills when completing the night shift independently, and at the same time they need to have the ability to communicate effectively with the patients, and the special environment and challenges make the night shift nurses not only improve their professional skills, but also establish a good nurse–patient relationship with the patients, which makes it easier for them to be recognized by the patients and thus perceive higher professional value; on the other hand, most of the respondents of the survey are nurses in tertiary hospitals, which are the centers of regional health care and not only have more generous professional compensation compared with other hospitals but also have more development opportunities and more opportunities to develop their professional skills. On the other hand, most of the survey respondents are nurses in tertiary general hospitals, which are the center of regional healthcare and not only have more generous professional compensation than other hospitals, but also have more opportunities and platforms for development, which enables nurses to obtain higher job satisfaction and work value by completing their own specialized work, and thus have a higher level of sense of occupational benefit.

In this study, there is a significant relationship between age and perceived occupational benefit, and the older the nurses, the higher the perceived occupational benefit, which is consistent with the study of Gao et al.^[28]^ The reasons for this were analyzed: on the one hand, the older the nurses were able to obtain more family support, and effective family support improves nurses’ positive perceptions of their careers, enabling them to devote themselves to their work in a more positive state, and thus to discover the value and benefits of their work^[29]^; on the other hand, with age, night nurses are more On the other hand, with age, night nurses become more experienced and are more familiar with the treatment and care of various diseases to provide better care for patients, and the increase in experience and competence can bring nurses a direct sense of self-confidence and fulfillment, which increases the sense of occupational benefit.

In this study, educational qualification is one of the main factors affecting the perception of occupational benefit, and the higher the educational qualification, the higher the level of occupational benefit for nurses. Previous studies have shown that highly educated nurses usually have more choices in their career development and are more likely to receive career advancement and promotion, which makes them more confident in their careers^[30]^; at the same time, highly educated nurses will have higher salary packages than nurses with low education, and the financial advantage can provide them with better quality of life and career satisfaction, thus increasing the level of career sense of gain.

In this study, whether or not they have worked in the fight against the COVID-19 is one of the main factors affecting the sense of occupational benefit, and nurses who have worked in the fight against the COVID-19 have a higher level of occupational benefit, which is consistent with the findings of Wang et al.^[31]^ Nurses play a very important role during COVID-19. Most of the nurses are in direct contact with patients and bear the responsibility of saving patients, and this sense of responsibility and mission will lead to a deeper knowledge and pride in their work^[32]^; in addition, during the COVID-19, nurses need to continue to learn and master new skills and knowledge, as well as to strengthen the communication with doctors, and organizations in order to cope with the challenges, thus broadening nurses’ professional skills and organizational and coordination abilities, and promoting their overall growth, which in turn increases the sense of occupational benefit.^[33]^

In this study, psychological resilience was positively correlated with the sense of occupational benefit (*R* = 0.767, *P* < .001), indicating that the higher the psychological resilience, the higher the sense of occupational benefit for night shift nurses. In this case, we further conducted multivariate hierarchical regression analyses to explore the extent to which psychological resilience ultimately influences the sense of occupational benefit. As shown in Table 5, in Model 1, age, education, and whether or not they have worked in the fight against the COVID-19 significantly influenced the sense of occupational benefit (*F* = 18.896, *P* < .001), contributing 14.9% of the total degree of variation in the sense of occupational benefit, which further indicated that age, education, and whether or not they had been involved in outbreak prevention and control had a limited effect on the sense of occupational benefit. In Model 2, the results showed that psychological elasticity could significantly affect the sense of occupational benefit (*F* = 135.268, *P* < .001), contributing about 45.0% of the total variance, which indicated that psychological elasticity was an important predictor variable of the sense of occupational benefit, further verifying the validity of Hypothesis 2 in agreement with the study of Liu et al.^[12]^ Analyzing the reasons, nurses with higher psychological resilience are able to show positive self-regulation when facing stressful work environments, maintain high energy, pay attention to their own physical and mental health, and demand timely help and support, and this ability of self-regulation and self-care not only helps nurses cope with work stress, but also helps to improve their overall quality of life and sense of well-being at work, so that they are able to experience their work and self-worth from the stress of. At the same time, with the continuous progress of medical technology and the updating of nursing concepts, nurses need to continuously learn and adapt to new knowledge and skills, and the higher the psychological resilience of nurses, the more quickly they can accept new knowledge and skills, and better adapt to changing environments and role requirements, so as to improve the efficiency of the work and quality of work, and to explore the meaning and value of work.^[34]^ The higher the psychological resilience, the more quickly nurses can accept new knowledge and skills, and better adapt to the changing environment and role requirements, so as to improve work efficiency and work quality, from which they can explore the meaning and value of their work.

**Table 5:** Multiple stratified regression analyses of factors influencing night shift nurses’ sense of occupational gain.

Variant	Model 1			Model 2		
	β	t	*P* value	β	t	*P* value
Control variables						
1. Age	8.312	4.079	.000	3.359	2.385	.017
2. Academic qualification	8.346	4.686	.000	3.586	2.908	.004
3. Whether or not they have worked in the fight against the COVID-19	‐9.215	‐6.096	.000	‐2.321	‐2.179	.030
Psychological resilience				0.859	28.303	.000
*F* (*P* value)		18.896 (<.001)			135.268 (<.001)	
*R* ^2^		0.157			0.604	
*R*^2^ change		0.149			0.599	

Dependent variable: presenteeism; *P* value was derived using multiple regression analysis, α = 0.05.

There are some limitations in this study due to the lack of time and energy. Firstly, this study only took part of the night shift nurses in some general hospitals in Sichuan Province to conduct the survey, the scope of the study is relatively limited, and the results obtained may be insufficiently representative; secondly, this study took the form of a network questionnaire survey, and the results of the relevant entries may be subjective; finally, this study was only a cross-sectional survey study, which could not observe the dynamic change characteristics of night shift nurses’ sense of occupational benefit. Finally, this study was only a cross-sectional survey study, which could not observe the dynamic change characteristics of night shift nurses’ sense of occupational benefit, and at the same time, there was insufficient evidence on the causal relationship between psychological flexibility and sense of occupational benefit. In future research, we will further expand the sample range and sample size, and conduct dynamic observation of night shift nurses’ sense of occupational benefit to find out the trend of occupational benefit, so as to lay the foundation for the subsequent development of corresponding interventions to improve the occupational benefit of night shift nurses.

## 5. Conclusion

The sense of occupational benefit of night nurses is at a medium-high level and needs to be further improved. Age, education, and whether or not they have participated in epidemic prevention and control are the influencing factors of night shift nurses’ sense of occupational benefit, and the psychological resilience of night shift nurses is closely related to their sense of occupational benefit. Therefore, we suggest that nursing managers should pay high attention to nurses with low levels of perceived occupational benefit and enhance the development of interventions for their perceived occupational benefit according to different characteristics.

## Author contributions

**Conceptualization:** Zhenfan Liu.

**Data curation:** Zhenfan Liu, Jijun Wu.

**Funding acquisition:** Zhenfan Liu.

**Investigation:** Zhenfan Liu, Xiaoting Yan, Cui Chen, Jing Lu.

**Methodology:** Zhenfan Liu, Xiaoting Yan.

**Writing – original draft:** Zhenfan Liu.

**Writing – review & editing:** Zhenfan Liu.
